# Constructing an extracellular matrix-related prognostic model for idiopathic pulmonary fibrosis based on machine learning

**DOI:** 10.1186/s12890-023-02699-8

**Published:** 2023-10-19

**Authors:** Hong Luo, Jisong Yan, Xia Zhou

**Affiliations:** Department of Tuberculosis and Respiratory, Hubei Clinical Research Center for Infectious Diseases, Wuhan Research Center for Communicable Disease Diagnosis and Treatment, Wuhan Jinyintan Hospital, Tongji Medical College of Huazhong University of Science and Technology, Chinese Academy of Medical Sciences, Joint Laboratory of Infectious Diseases and Health, Wuhan Institute of Virology and Wuhan Jinyintan Hospital, Chinese Academy of Sciences, Wuhan, 430023 China

**Keywords:** IPF, Extracellular matrix, Bioinformatics, Immune infiltration, Prognosis

## Abstract

**Background:**

Idiopathic pulmonary fibrosis (IPF) is a chronic and progressive interstitial lung disease. Multiple research has revealed that the extracellular matrix (ECM) may be associated with the development and prognosis of IPF, however, the underlying mechanisms remain incompletely understood.

**Methods:**

We included GSE70866 dataset from the GEO database and established an ECM-related prognostic model utilizing LASSO, Random forest and Support vector machines algorithms. To compare immune cell infiltration levels between the high and low risk groups, we employed the ssGSEA algorithm. Enrichment analysis was conducted to explore pathway differences between the high-risk and low-risk groups. Finally, the model genes were validated using an external validation set consisting of IPF cases, as well as single-cell data analysis.

**Results:**

Based on machine learning algorithms, we constructed an ECM-related risk model. IPF patients in the high-risk group had a worse overall survival rate than those in the low-risk group. The model’s AUC predictive values were 0.786, 0.767, and 0.768 for the 1-, 2-, and 3-year survival rates, respectively. The validation cohort validated these findings, demonstrating our model’s effective prognostication. Chemokine-related pathways were enriched through enrichment analysis. Moreover, immune cell infiltration varied significantly between the two groups. Finally, the validation results indicate that the expression levels of all the model genes exhibited significant differential expression.

**Conclusions:**

Based on CST6, PPBP, CSPG4, SEMA3B, LAMB2, SERPINB4 and CTF1, our study developed and validated an ECM-related risk model that accurately predicts the outcome of IPF patients.

**Supplementary Information:**

The online version contains supplementary material available at 10.1186/s12890-023-02699-8.

## Background

Idiopathic pulmonary fibrosis (IPF), an interstitial lung disease, is distinguished by rarity, persistent progression, and fibrosis, with an etiology and pathogenesis that remains incompletely understood [1]. Smoking, exposure to dust, certain viral infections (such cytomegalovirus and Epstein-Barr virus), and gastroesophageal reflux are risk factors for IPF. Furthermore, certain mutations in the telomerase gene may be associated with familial IPF [2]. IPF can occur in the general population at a rate of between 1/100,000 and 45/100,000 and typically occurs in individuals of middle age or older, with a greater susceptibility among elderly men who have a significant smoking history (> 20 pack-years) [3]. Pirfenidone and nintedanib are currently the primary treatments for IPF; however, they cannot prevent disease progression, and further research is necessary to determine whether these drugs are suitable for severe IPF patients [4]. Hence, there is a pressing need to investigate the pathophysiology of IPF and established new predictive signatures associated with its prognosis.

Extracellular matrix (ECM) is a critical component of tissues and organs, playing an essential role in the survival of multicellular organisms, and serving as a key regulator of cellular behavior. Comprising over 300 different proteins, including collagens (primarily types I and III), elastin, glycoproteins, and proteoglycans such as hyaluronic acid and fibronectin, these molecules influence cell adhesion, migration, and differentiation [5]. Initially considered a simple scaffold supporting the anatomical structure of the lungs and providing structural support for the airways, the ECM has been shown to be a dynamic structure capable of fine-tuning through varying degrees of tissue differentiation, synthesis, deposition, degradation, and resorption, serving as a component of normal tissue healing and pathological processes [6]. In IPF, a dynamic imbalance between collagen synthesis and degradation leads to excessive ECM deposition in the lung interstitium [7]. Type III collagen levels rise in the early stages of IPF fibrosis, while type I collagen levels rise and type III collagen levels fall in the late stages. This change may be due to a decrease in collagenase activity and an increase in enzyme inhibitor activity leading to a decreased collagen breakdown rate [8]. Laminin is a large molecular non-collagenous glycoprotein unique to the transparent layer of the basement membrane that affects cell adhesion, growth, and differentiation. In the late stages of IPF, laminin increases significantly, attracting fibroblasts and inflammatory cells to accumulate in the basement membrane, damaging lung tissue, stimulating fibroblasts and epithelial cells to secrete collagen, and thus leading to lung fibrosis [9]. Hyaluronic acid is a large molecular glycosaminoglycan in the lung that is synthesized in large quantities by fibroblasts under the influence of pathogenic factors such as oxygen free radicals, and has a certain role in pulmonary fibrosis [10]. These findings imply that ECM may influence the development and prognosis of IPF. However, the correlation between ECM and IPF prognosis is not sufficiently supported by the available data. Therefore, the establishment of an IPF prognosis model containing ECM-associated genes (EAGs) is of great significance.

In this study, we aimed at exploring the relationship between EAGs and prognosis in IPF. We established an ECM-related prognosis model through LASSO and Cox regression analysis, and verified its reliability using the GEO cohort. Furthermore, we studied the interaction between this model and immune cells. Our findings reveals the potential value of EAGs in treating IPF patients and help us comprehend the correlation between ECM and IPF prognosis even better.

## Methods

### Screening model genes based on LASSO, random forest and support vector machine algorithms

We retrieved 1,062 EAGs from the previously published literature [11]. To further elucidate the functionality of these genes in the progression of idiopathic pulmonary fibrosis (IPF), we conducted an analysis of the GSE70866 dataset. This dataset comprises gene expression profiles derived from bronchoalveolar lavage (BAL) cells of patients diagnosed with IPF [12]. BAL samples were collected from 176 patients at three different medical centers, namely Freiburg, Siena, and Leuven (Tables 1 and 2). Among these patients, the training set consisted of 112 individuals diagnosed with IPF from the Freiburg and Siena cohorts, which were analyzed using the GPL14550 platform. The validation set comprised 64 IPF patients from the Leuven cohort, and their data were analyzed using the GPL17077 platform. Additionally, twenty healthy individuals were recruited as controls at the University Medical Center Freiburg in Germany. These control subjects were deemed healthy based on pulmonary function tests and clinical examinations, ruling out any lung diseases. The diagnosis of IPF was established by a multidisciplinary board at each institution, adhering to the criteria set forth by the American Thoracic Society/European Respiratory Society, and subsequently aligned with recent guidelines [13–15]. Only patients displaying idiopathic UIP and a HRCT consistent with a “definite” or “possible” UIP pattern were included in the study. For patients categorized as having a “possible” UIP pattern on HRCT, a histological confirmation of UIP was required. Lung biopsies were obtained from 33% of the affected subjects. Pulmonary function tests were conducted in all three centers using standardized methodologies, following the recommendations outlined by the American Thoracic Society/European Respiratory Society, employing a body plethysmograph [16]. None of the patients had received pirfenidone or nintedanib prior to undergoing BAL examination. However, during the follow-up period, the patients received various treatment regimens, including corticosteroids, azathioprine, N-acetylcysteine, and pirfenidone. Subsequently, we performed gene screening using LASSO, random forest and support vector machine algorithms, and then took the intersection set to obtain the model genes. Subsequently, ROC analysis and differential expression analysis were performed on these model genes.

**Table 1:** Baseline Characteristics of IPF patients and controls in training cohort

Characteristics	Freiburg cohort (*n* = 62)	Siena cohort (*n* = 50)	Control cohort (*n* = 20)
Age, yr	67.4 ± 9.1	68.7 ± 11.2	61.9 ± 7.6
Male sex, %	85	80	75
FVC % predicted value, %	66 ± 20	67 ± 23	96 ± 19
DLCO			
Percent predicted value, %	44 ± 16	40 ± 15	–
Could not perform DlCO, n	7	12	–
Deaths, n (%)	45 (73)	31 (62)	–
Transplants, n (%)	3 (5)	4 (8)	–
Median observation time, mo	20	16	–
Smoking status, %			
Never smoked	42	34	30
Former smoker	56	64	70
Current smoker	2	2	0
HRCT UIP, n (%)			
Definite	43 (69)	38 (76)	–
Possible	19 (31)	12 (24)	–
HRCT emphysema present, n	5 (8)	3 (6)	–
GAP index, n (%)			
Stage I	17 (27)	14 (28)	–
Stage II	32 (52)	20 (40)	–
Stage III	13 (21)	16 (32)	–

*Abbreviations*: *HRCT* high-resolution computed tomography, *IPF* idiopathic pulmonary fibrosis, *UIP* usual interstitial pneumonia. Data are mean ± SD unless otherwise indicated

**Table 2:** Baseline Characteristics of IPF patients in validation cohort

Characteristics	Leuven cohort (*n* = 64)
Age, yr	68.2 ± 8.5
Male sex, %	80
FVC % predicted value, %	78 ± 18
DLCO	
Percent predicted value, %	45 ± 12
Could not perform DlCO, n	1
Deaths, n (%)	24 (38)
Transplants, n (%)	3 (5)
Median observation time, mo	18
Smoking status, %	
Never smoked	23
Former smoker	70
Current smoker	6
HRCT UIP, n (%)	
Definite	53 (83)
Possible	11 (17)
HRCT emphysema present, n	16 (25)
GAP index	
Stage I	25 (39)
Stage II	31 (48)
Stage III	8 (13)

*Abbreviations*: *HRCT* high-resolution computed tomography, *IPF* idiopathic pulmonary fibrosis, *UIP* usual interstitial pneumonia. Data are mean ± SD unless otherwise indicated

### Development and validation the ECM-related model

Firstly, we carried out a multivariate Cox regression analysis on the selected model genes and constructed a risk score to assess the prognosis of IPF by calculating the formula: $\mathrm{risk score}={\sum }_{i}^{n}Ci\times Ei$, where n represents the number of genes, C represents the regression coefficient, and E represents the gene expression level. According to the median risk score, we split IPF cases into high- and low-risk score groups. We next used survival analysis to assess the correlation between overall survival and the ECM-related risk model. The prognostic value of the ECM-related risk model was evaluated through time-dependent ROC curve and the corresponding area under the curve (AUC), and the efficacy of the prognostic model was assessed using the “survminer” and “timeROC” packages. Finally, we developed a nomogram with the training set, applied the “rms” package to forecast the 1-, 2-, and 3-year survival rates of IPF patients, and assessed the nomogram’s prediction efficiency using the calibration curve. The accuracy of the ECM-related risk model’s prediction ability for the prognosis of IPF was validated using the validation set. The findings of this study offer an innovative and trustworthy way for determining an IPF patient's prognosis.

### Immune infiltration level analysis

In this study, the ssGSEA algorithm was applied to dissect the gene expression profiles of high and low risk groups of patients, resulting in the identification of 28 immune cell subtypes and their scores in clinical samples [17]. In order to assess the relationship between these immune cells and ECM-related model genes, we also calculated the correlation between them.

### Enrichment analysis

To elucidate the molecular mechanisms and pathway differences between high and low-risk groups, we conducted gene ontology (GO), Kyoto Encyclopedia of Genes and Genomes (KEGG), and gene set enrichment analysis (GSEA) using the “clusterProfiler” package [18, 19]. These analyses provide a deeper understanding of the expression patterns and functional enrichment of various biological processes and pathways in high-risk and low-risk patients. Through enrichment analysis, we can further investigate the roles of these pathways and biological processes in the occurrence and progression of IPF, aiming at providing new ideas and methods for IPF treatment.

### Validation of model genes based on external validation set and single cell analysis

We incorporated the external validation set GSE28042 to validate the expression levels of the seven model genes [20, 21]. Furthermore, we extended our validation efforts by analyzing the expression of these model genes in mice with idiopathic pulmonary fibrosis (IPF) using single-cell analysis. To conduct this analysis, we utilized SPEED, an online tool for single-cell multi-omics analysis that integrates diverse datasets encompassing evolutionary, developmental, and disease-related information from over 120 species [22]. The single-cell sequencing data can be accessed in the GEO database under the dataset ID GSE129605 [23]. Initially, we employed the tSNE algorithm to reduce the dimensionality of the single-cell expression data and cluster the cells based on their expression profiles. Cell types were defined based on classical cell markers. Subsequently, heatmaps were generated to compare the expression levels of the model genes across different cell types.

## Results

### Selection of model genes

**Fig. 1:**
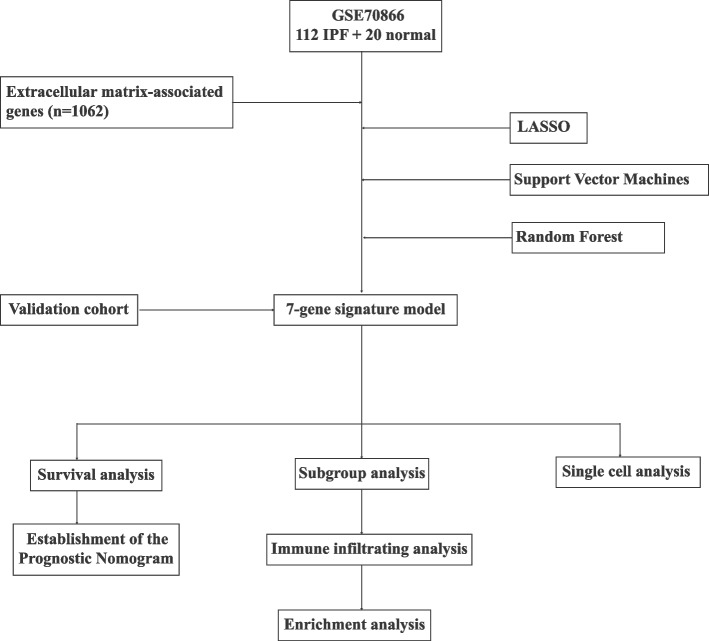
Workflow diagram of this study

**Fig. 2:**
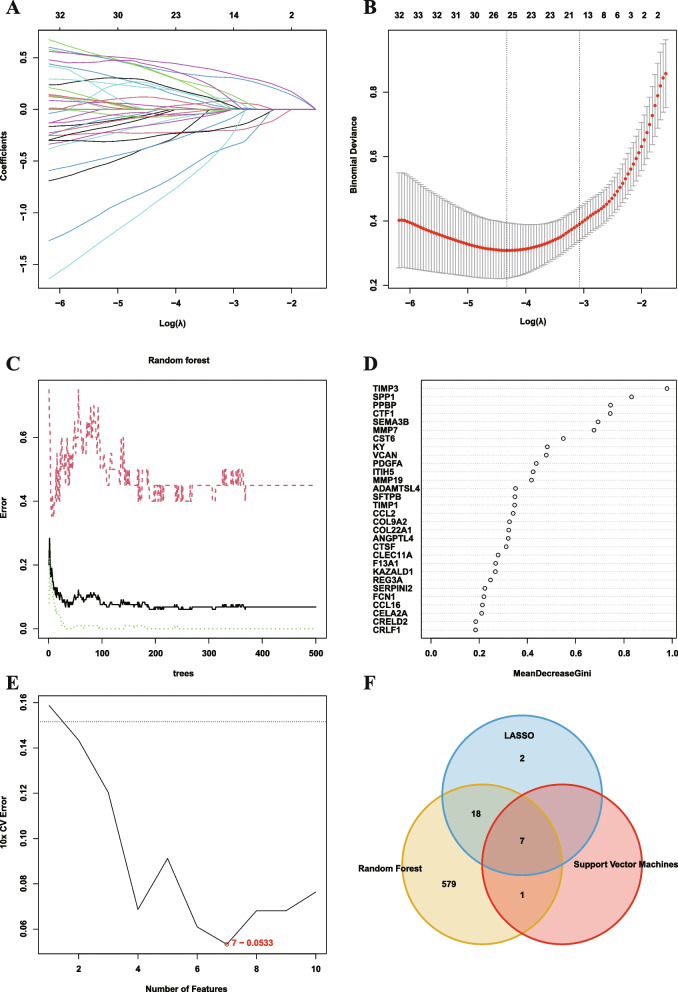
Screening of critical signatures via multiple machine-learning algorithms. **A**-**B** Screening of critical signatures via LASSO regression. **C**-**D** Screening of critical signatures via random forest algorithm. **E** Screening of critical signatures via support vector machines algorithm. **F** Intersection of critical signatures via multiple machine-learning algorithms

**Fig. 3:**
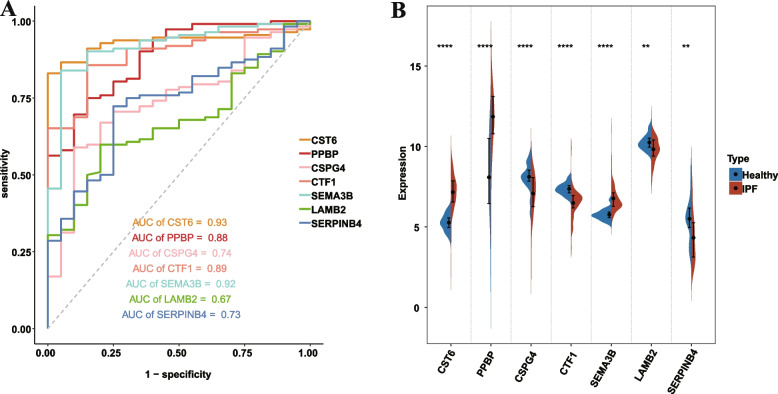
ROC and differential expression analysis of model genes. **A** ROC analysis of model genes. **B** Differential expression analysis of model genes. *p < 0.05, **p < 0.01, ***p < 0.001, and ns, no significance

Figure 1 illustrates the workflow. Using LASSO algorithm (Fig. 2A-B), random forest algorithm (Fig. 2C-D) and support vector machine algorithm (Fig. 2E), we screened 27 EAGs, 605 EAGs and 8 EAGs respectively. Then, a total of 7 model genes were obtained by taking the intersection set (Fig. 2F). ROC analysis showed that these 7 model genes could well discriminate between IPF and healthy individuals (Fig. 3A). Furthermore, they were all differentially expressed between IPF and healthy individuals (Fig. 3B).

### Determination of prognostic risk model of EAGs in IPF

**Fig. 4:**
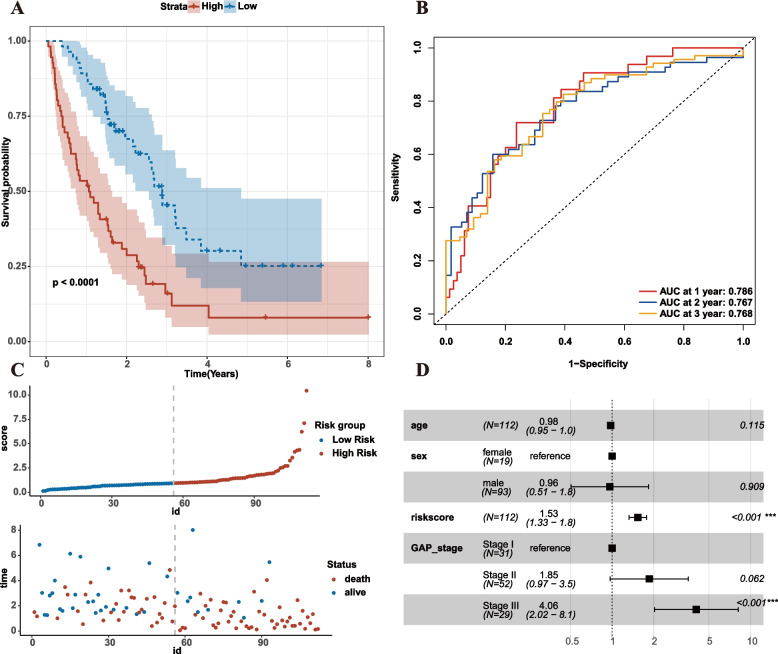
Risk score analysis of the ECM-related prognostic model in the GSE70866 training cohort. **A** Survival curve of high-risk score and low-risk score groups. **B** Risk plot distribution and survival status. **C** ROC curves evaluated the efficiency for predicting 1-, 2-, and 3-year survival. **D** Multivariate cox analysis of riskscore in GSE70866

We applied multivariate Cox analysis to establish a prognostic risk model consisting of 7 EAGs. The following formula determines the risk score: Risk score = -7.4224611 + 0.40795439 * CST6 + 0.10526373 * PPBP—0.17336452 * CSPG4 + 0.59799764 * SEMA3B + 0.013215737 * LAMB2—0.024697116 * SERPINB4 + 0.063153152 * CTF1. Discrepancies in survival rates between high and low risk subgroups were observed in the survival plots (*P* < 0.001, C-index, 0.707; 95% CI, 0.649–0.766), with the low-risk subgroup having a higher survival rate (Fig. 4A). The AUC predictive values of this model were 0.786, 0.767, and 0.768, which could be utilized to evaluate the 1-, 2-, and 3-year survival rates of IPF patients (Fig. 4B). The survival status display and risk distribution plot demonstrated that patients with low risk scores had considerably longer survival times than those with high risk scores (Fig. 4C). Multivariate Cox analysis indicated, after adjusting for GAP stage confounding factors, the risk score could serve as an independent and effective marker for evaluating the prognosis of IPF patients (Fig. 4D).

### Verification of the ECM-related prognostic model

**Fig. 5:**
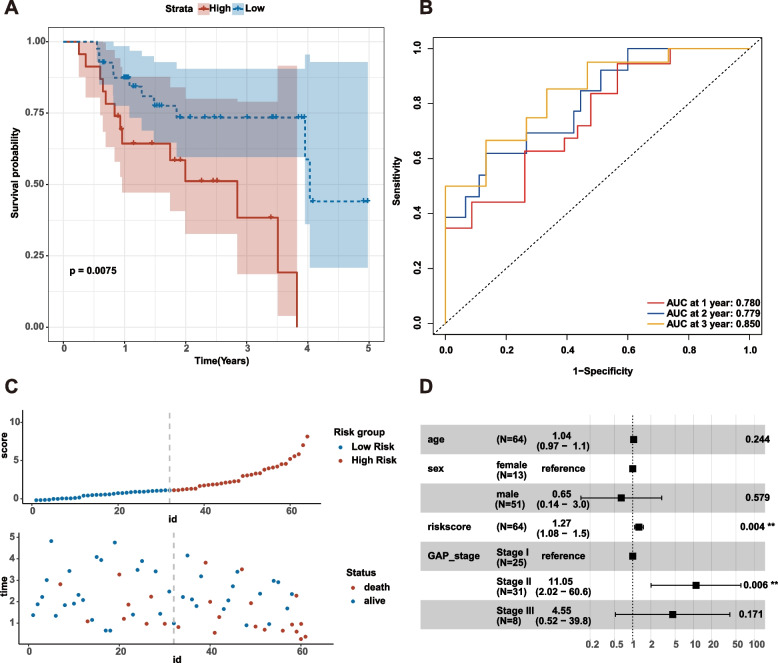
Validation of the ECM-related prognostic model in GSE27957. **A** Survival curve of high-risk score and low-risk score groups. **B** Risk plot distribution and survival status. **C** ROC curves evaluated the efficiency for predicting 1-, 2-, and 3-year survival. **D** Multivariate cox analysis of riskscore in GSE27957

This study used a validation cohort to verify the ECM-related prognostic model. Discrepancies in survival rates between high and low risk subgroups were also observed in the survival plots (*P* = 0.0075, C-index, 0.727; 95% CI, 0.619–0.834) (Fig. 5A). Based on the median risk score, IPF patients were split into high and low risk subgroups, and those in the high-risk category had lower survival status (Fig. 5C). The validation dataset's AUC values for the ROC curves for 1-year, 2-year, and 3-year survival were, respectively, 0.780, 0.779, and 0.850 (Fig. 5B). After adjusting for GAP stage confounding factors,the risk score can be utilized as a reliable independent predictor for assessing the prognosis of IPF patients, according to multivariate Cox analysis (Fig. 5D).

### Construction of nomogram based on seven EAGs

**Fig. 6:**
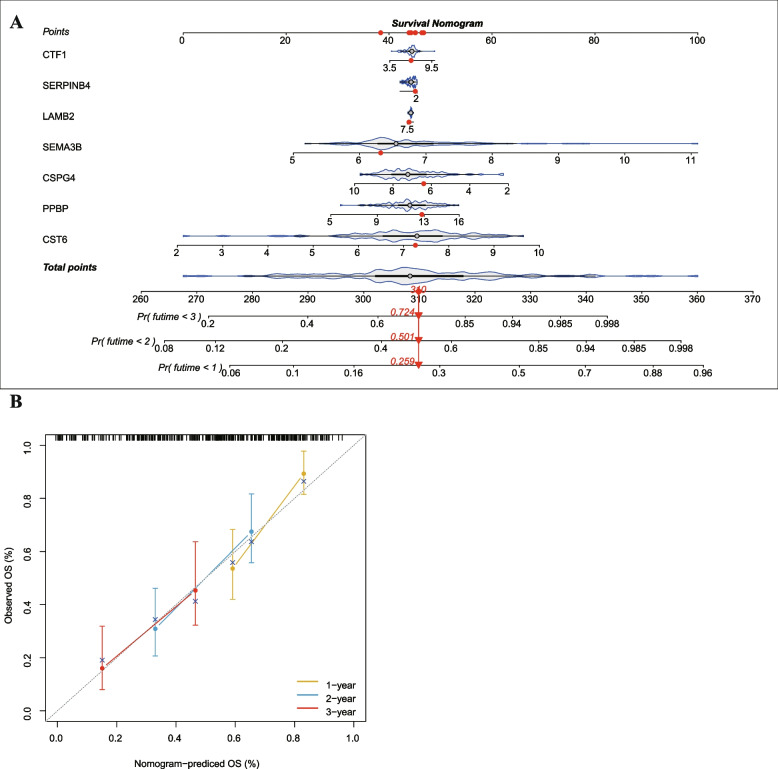
Construction and evaluation of a nomogram for predicting 1-, 2-, and 3-year overall survival rates of IPF patients. **A** Nomogram for predicting 1-, 2-, and 3-year overall survival of IPF patients. **B** Calibration curves showing the probability of 1-, 2-, and 3-year overall survival between the prediction and the observation

To build a quantitative way for forecasting the prognosis of IPF patients, this study screened eight EAGs and utilized them to establish a predictive model (Fig. 6A). By analyzing the calibration curve, we found a high degree of consistency between the predicted and observed values (Fig. 6B). Therefore, this predictive model based on the seven EAGs exhibited good accuracy and can forecast the outcome of IPF patients.

### The relationship between the ECM-related prognostic model and immune cell in IPF

**Fig. 7:**
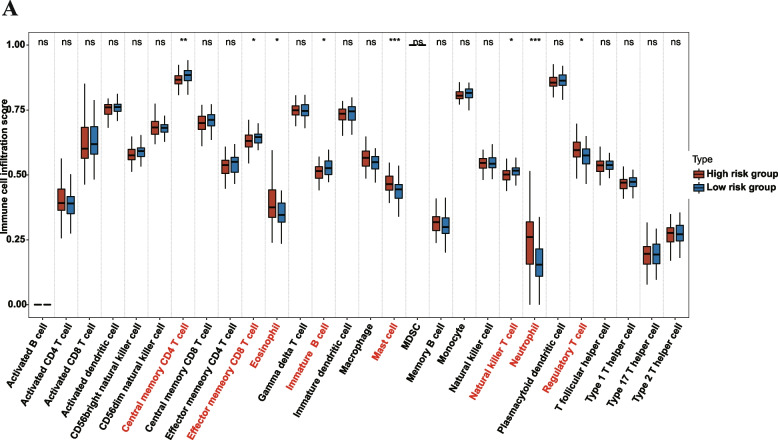
Relationship between ECM-related prognostic model and immune cell infiltration in IPF. **A** The difference of 28 immune cells between the high-risk score group and the low-risk score group. *p < 0.05, **p < 0.01, ***p < 0.001, and ns, no significance

The discrepancy in levels of 28 immune cell infiltrations between the groups with high and low risk scores was calculated utilizing the ssGSEA algorithm (Fig. 7A). The findings revealed IPF patients with high-risk score exhibited higher levels of infiltrated activated CD4T cells, CD56dim natural killer cells, eosinophils, macrophages, neutrophils, regulatory T cells, and T follicular helper cells, and lower levels of central memory CD4 T cells infiltration. The relationship between certain immune cells and EAGs was then investigated (Fig. S1A-G).

### ECM-related prognostic model enrichment analysis

**Fig. 8:**
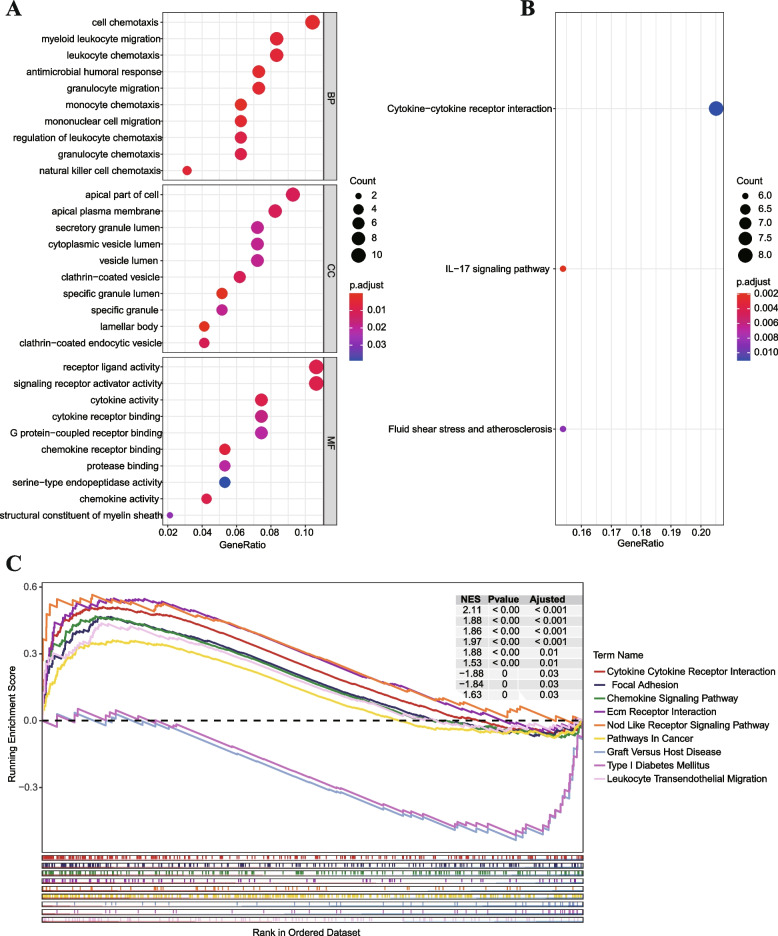
Functional enrichment analysis of ECM-related prognostic model between high-risk and low-risk score groups. **A** GO enrichment. **B** KEGG pathway analyses. **C** GSEA analyses

In order to elucidate the biological activities and pathways related to the ECM-related model, we carried out GO and KEGG analyses. According to the GO enrichment results, the high-risk score group was connected with pathways relevant to cell chemotaxis, including cell chemotaxis, leukocyte chemotaxis, monocyte chemotaxis, and lymphocyte chemotaxis (Fig. 8A). KEGG pathway analysis showed that cytokine − cytokine receptor interaction and IL − 17 signaling pathway were enriched (Fig. 8B). GSEA analysis revealed that patients in the high-risk score group exhibited downregulation of the leukocyte transendothelial migration pathway, while upregulation of the chemokine signaling pathway, cytokine − cytokine receptor interaction pathway, and focal adhesion pathway (Fig. 8C).

### Verification of the model genes in external validation set and single-cell analysis

**Fig. 9:**
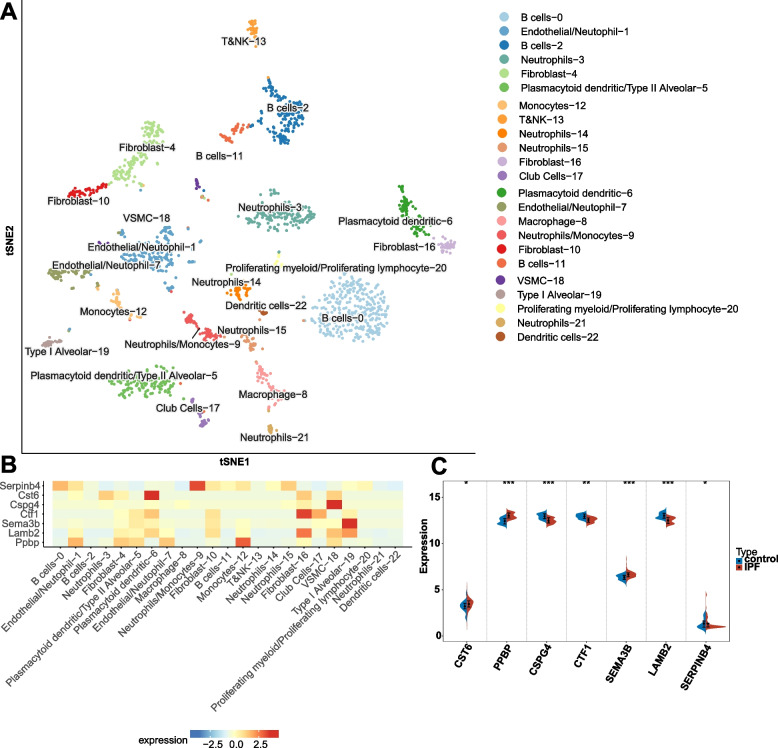
Validation of model genes expression in external validation set and single-cell data. **A** The tSNE plot shows the cell types identified in IPF with different colors. **B** Heatmap depicts model genes expression across major cell types. **C** Differential expression analysis of model genes in external validation set. *p < 0.05, **p < 0.01, ***p < 0.001, and ns, no significance

We investigated the correlation between the expression of the model genes and various cell populations. Through clustering analysis, we identified 22 distinct cell clusters, which were further consolidated into 14 cell populations based on the expression of marker genes. These populations encompassed B cells, Endothelial/Neutrophil cells, Fibroblast cells, Plasmacytoid dendritic/Type II Alveolar cells, Macrophage cells, Neutrophils/Monocytes, Monocytes, T&NK cells, Neutrophils, Club Cells, VSMC (vascular smooth muscle cells), Type I Alveolar cells, Proliferating myeloid/Proliferating lymphocyte cells, and Dendritic cells (Fig. 9A). Notably, our findings unveiled that Serpinb4 exhibited predominant expression in Neutrophils/Monocytes, Cst6 exhibited predominant expression in Plasmacytoid dendritic cells, and Cspg4 exhibited predominant expressiosn in VSMC (Fig. 9B). Moreover, all seven model genes demonstrated differential expression between IPF and control samples, aligning with the previous findings (Fig. 9C).

## Discussion

IPF is a disease with a currently incompletely elucidated etiology that poses a substantial global socioeconomic burden. Reducing morbidity and mortality from IPF requires early identification, prevention, and intervention of relevant risk factors. For predictive diagnosis, focused prevention, and individualized treatment of the disease, it is thought to be useful to screen for putative susceptibility genes and uncover their molecular mechanisms in IPF. In this work, we constructed an ECM-related risk model utilizing LASSO, Random forest and Support vector machines algorithms to forecast the prognosis of IPF patients. The results indicate this model, based on seven genes CST6, PPBP, CSPG4, SEMA3B, LAMB2, SERPINB4 and CTF1, is a reliable predictor of the survival rate of IPF patients. The model was validated in an independent dataset from the GEO cohort, GSE27957, which confirmed its good performance. In addition, the results of ROC analysis indicated that these seven genes could be used as biomarkers of IPF with high AUC values.

The identification of these seven EAGs is consistent with earlier studies, which found that the ECM is closely associated with the development of IPF. With the exception of CST6, SEMA3B, LAMB2, SERPINB4 and CTF1, the remaining two genes have all been linked to the progression of IPF. PPBP (pro-platelet basic protein) is a gene that encodes a cytokine protein called CTAP-III, which stimulates the formation and maturation of white blood cells, red blood cells, and platelets [24]. The results of two previous studies have both indicated an upregulation of PPBP expression in the bronchoalveolar lavage fluid of IPF patients, which is consistent with our own findings. However, studies exploring the precise mechanisms of PPBP in IPF are currently lacking [25, 26]. CSPG4 (Chondroitin Sulfate Proteoglycan 4), also known as NG2 or MCSP, is a transmembrane proteoglycan that is found on the outer layers of various cell types, including pericytes, glial cells, and melanoma cells. It is involved in cell adhesion, migration, and proliferation, as well as regulating the signaling pathways involved in these processes [27]. CSPG4 is a marker for pericytes, which are cells that play a role in maintaining the stability and function of blood vessels. In human lung pericytes, CSPG4 is expressed along with PDGFRB. These cells can change into myofibroblast-like cells, which are involved in the formation of fibrotic tissue, when they are driven by TGF- signaling. Fibroblastic foci, which are areas of fibrosis in the lungs, also express PDGFRB and CSPG4. Therefore, myofibroblasts may emerge from pericytes stimulated by TGF- signaling in fibroblastic foci, contributing to the development of pulmonary fibrosis. However, if the pericyte-myofibroblast transition is suppressed, it could potentially mitigate pulmonary fibrosis [28]. In summary, these data provide new insights into the roles of PPBP and CSPG4 in IPF, which lay the foundation for targeted prevention, progression tracking, prognosis evaluation, and individualized medicine. Furthermore, these findings could advance the development of treatment practices based on PPBP and CSPG4 targeting strategies.

In addition, enrichment analysis revealed that comparing the high-risk group to the low-risk group, chemokine-related pathways were considerably enriched in the high-risk group. It is well recognized that chemokines are crucial for the attraction and activation of immune cells, which may help to cause IPF. Moreover, GSEA analysis showed enrichment in the pathway for chemokine signaling, further supporting the importance of this pathway in IPF. KEGG pathway analysis showed that IL-17 pathway plays an essential part in the pathological mechanism of IPF. IL17 is secreted by CD4 + T cells and can induce the production and excretion of pro-inflammatory cytokines such as IL-6, IL-8, and G-CSF in epithelial cells, endothelial cells, and fibroblasts. This family includes six ligands (IL-17A, IL-17B, IL-17C, IL-17D, IL-17E, and IL-17F) and five receptors (IL-17RA, IL-17RB, IL-17RC, IL-17RD, and IL-17RE) [29]. Currently, Studies have revealed that IL-17A, IL-17B, and IL-17E are all involved in the development and promotion of pulmonary fibrosis [30]. Specifically, IL-17A is important in the development and progression of fibrosis induced by bleomycin and IL-1beta, while IL-17B directly induces the production of proinflammatory and profibrotic genes [31]. Elevated levels of IL-6 and IL-17 have been related to pulmonary fibrosis, and the elements of the IL-17A signaling cascade may serve as possible treatment targets for the treatment of fibroproliferative lung disorders [32]. Additionally, infection with bacteria can cause AE-IPF, and IL-17 may be a potential therapeutic target for this condition [33]. Finally, IL-17A also worsens type II alveolar epithelial cells’ ability to maintain mitochondrial homeostasis, which contributes to lung fibrosis [34].

Our findings also showed that the immune cell infiltration was different between the high and low risk subgroups. According to prior research revealing that immunological dysregulation is a crucial aspect of the disease, these findings imply that the immune response may be implicated in the etiology of IPF.

One limitation of our study is that it is based on retrospective data from public database, therefore, prospective studies are required to validate our results. In addition, the precise relationship between EAGs and IPF remains to be evaluated and validated in experimental studies because we have no access to clinical specimens of IPF.

## Conclusions

In conclusion, our study offers fresh perspectives on the function of ECM in IPF and identifies a new ECM-related risk model for forecasting the survival rate of IPF patients. These findings aid to a deeper comprehension of the association between EAGs and the prognosis of IPF and may provide a basis for developing personalized therapies for IPF patients. Further studies are required to validate the clinical usefulness of our ECM-related risk model and to investigate the underlying mechanisms by which these genes contribute to the pathogenesis of IPF.

### Supplementary Information

**Additional file 1.**

## Data Availability

The datasets generated and analysed during the current study are available in GEO (https://www.ncbi.nlm.nih.gov/geo), which are public functional genomics data repositories.
