# Supplementary figures and images for: Constructing an extracellular matrix-related prognostic model for idiopathic pulmonary fibrosis based on machine learning
Source: BMC Pulm Med. 2023 Oct 19;23:397. doi: 10.1186/s12890-023-02699-8 (PMC10585847; doi:10.1186/s12890-023-02699-8)

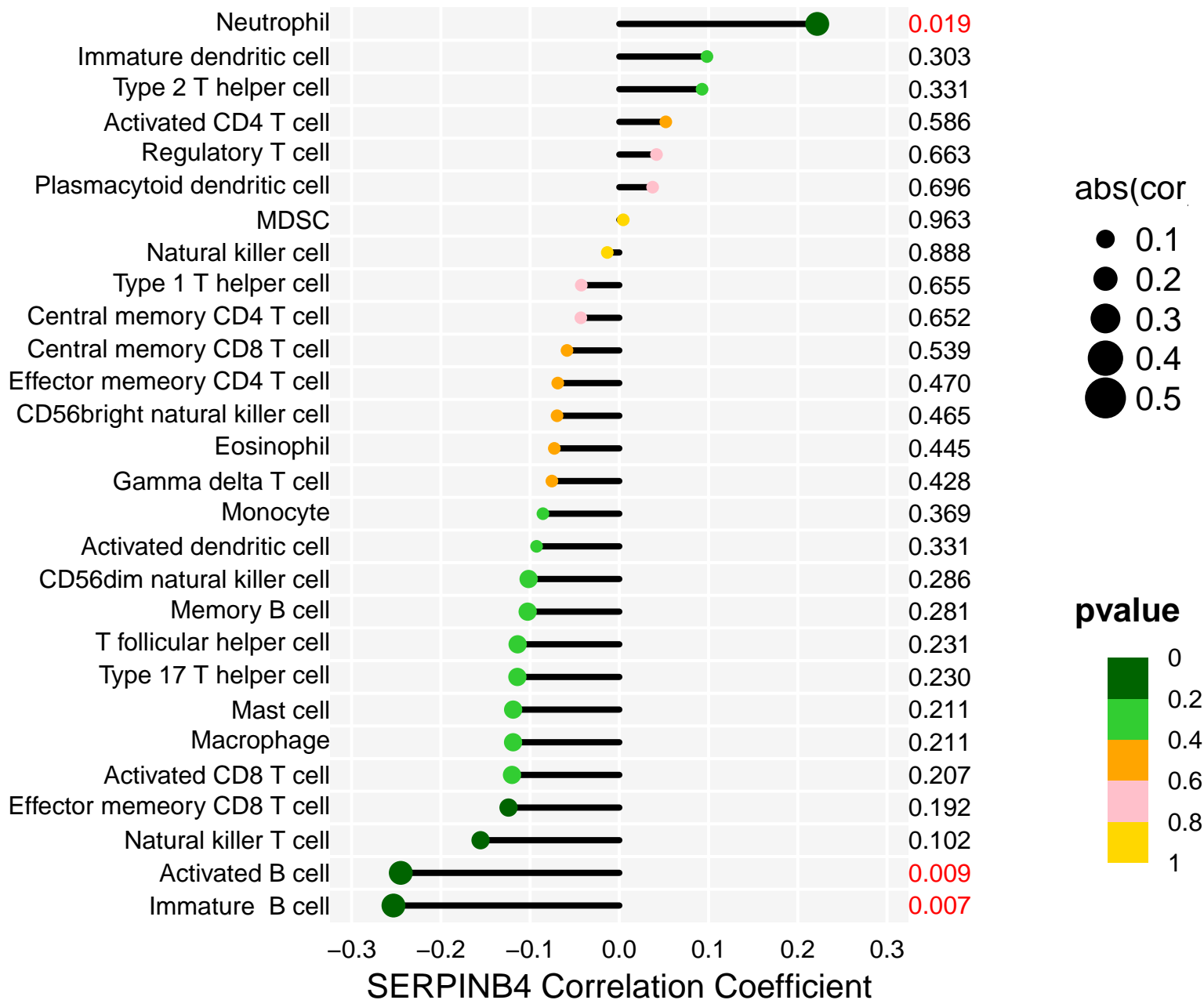

Supplement: Supplementary file 1 — Additional file 1. [file 12890_2023_2699_MOESM1_ESM.zip › 12890_2023_2699_MOESM1_ESM/Fig S1A_ESM.pdf]

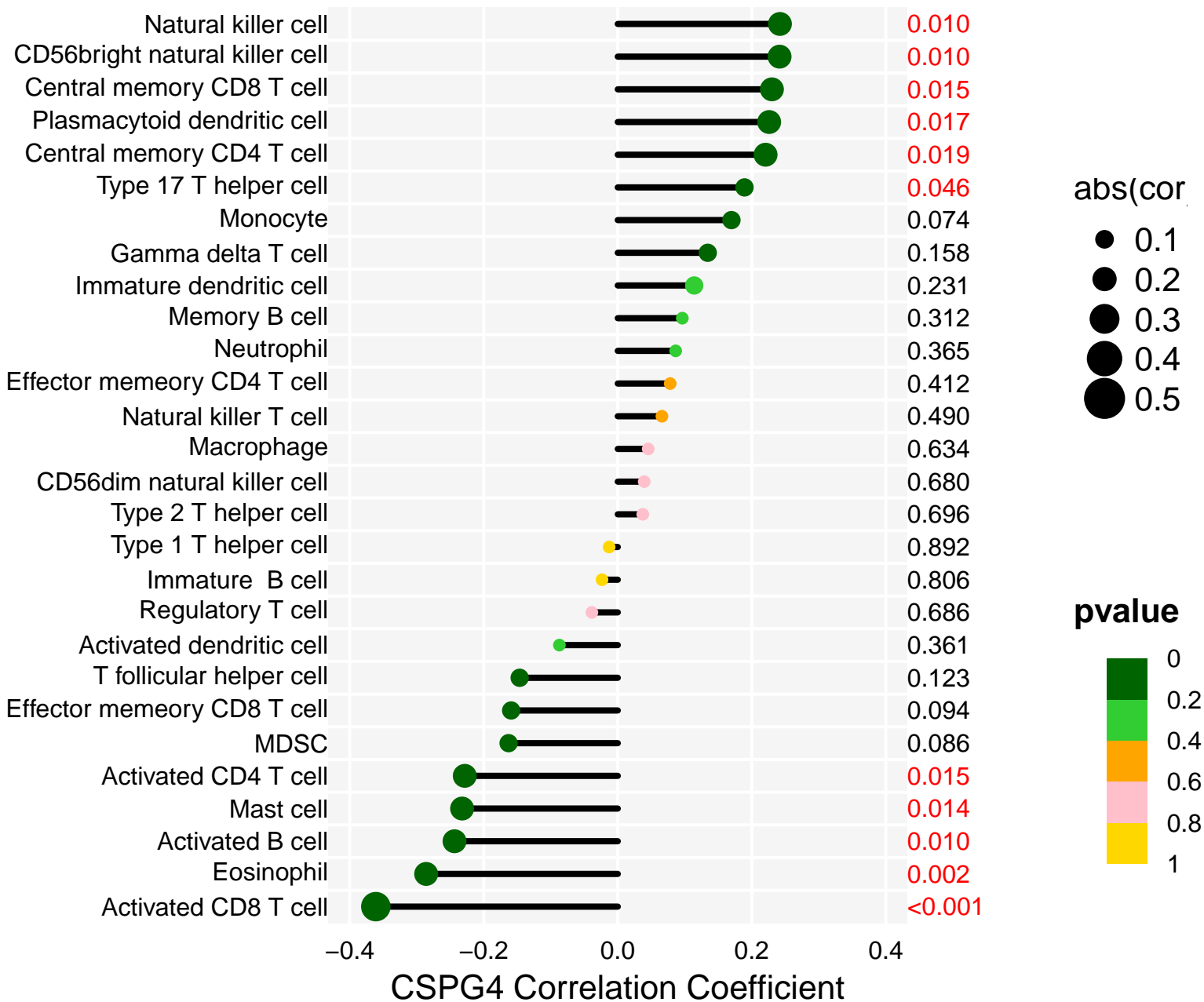

Supplement: Supplementary file 1 — Additional file 1. [file 12890_2023_2699_MOESM1_ESM.zip › 12890_2023_2699_MOESM1_ESM/Fig S1B_ESM.pdf]

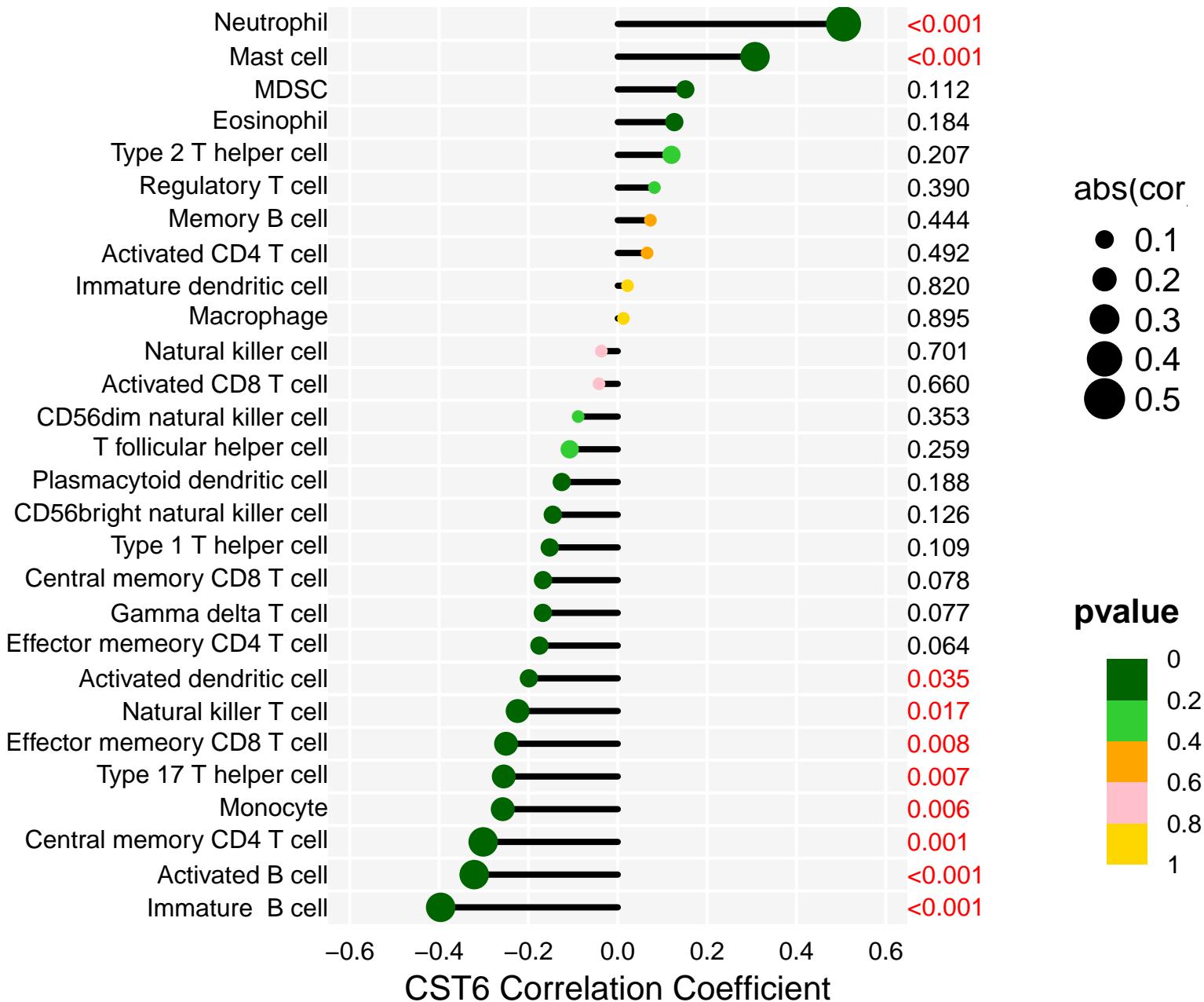

Supplement: Supplementary file 1 — Additional file 1. [file 12890_2023_2699_MOESM1_ESM.zip › 12890_2023_2699_MOESM1_ESM/Fig S1C_ESM.pdf]

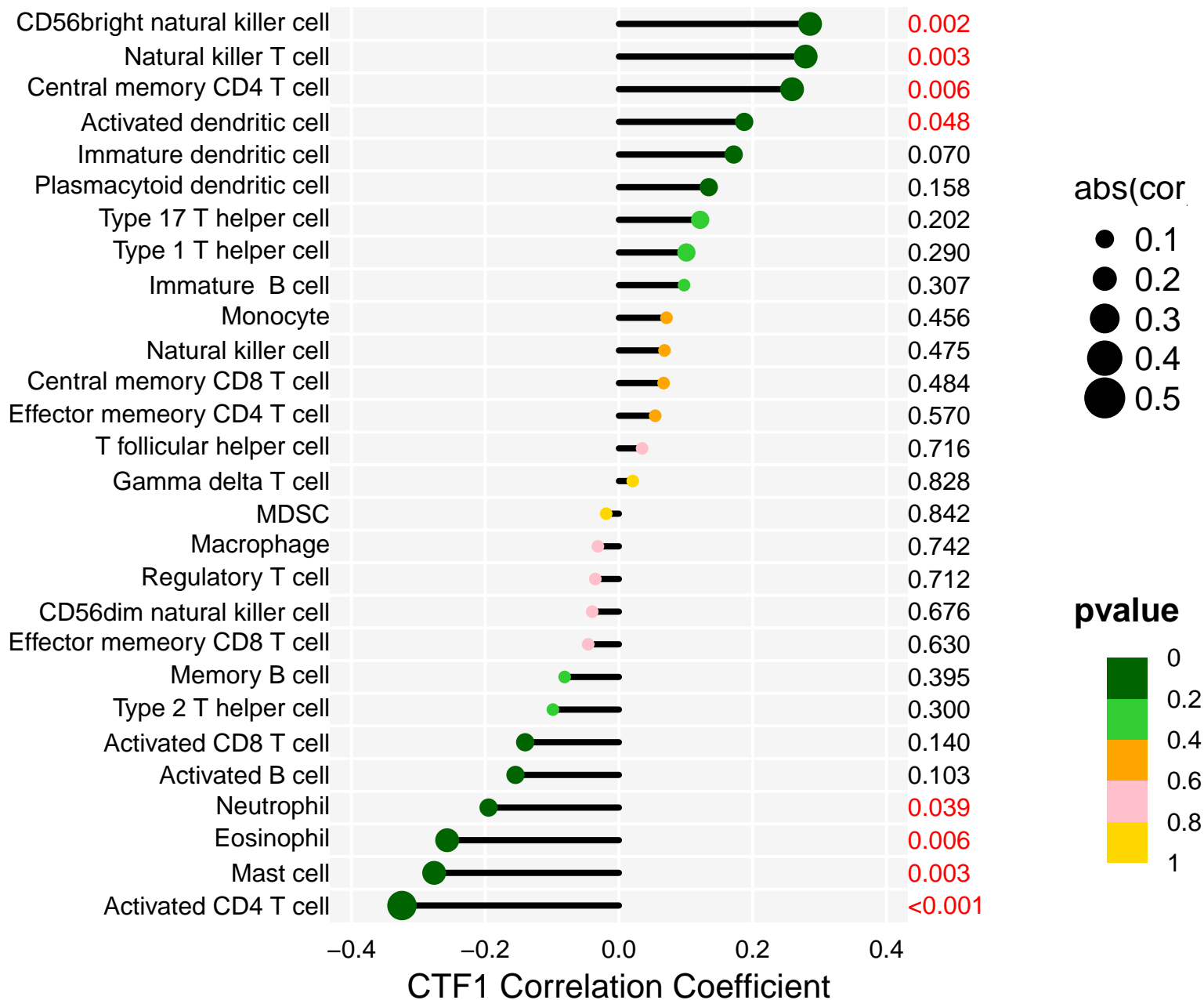

Supplement: Supplementary file 1 — Additional file 1. [file 12890_2023_2699_MOESM1_ESM.zip › 12890_2023_2699_MOESM1_ESM/Fig S1D_ESM.pdf]

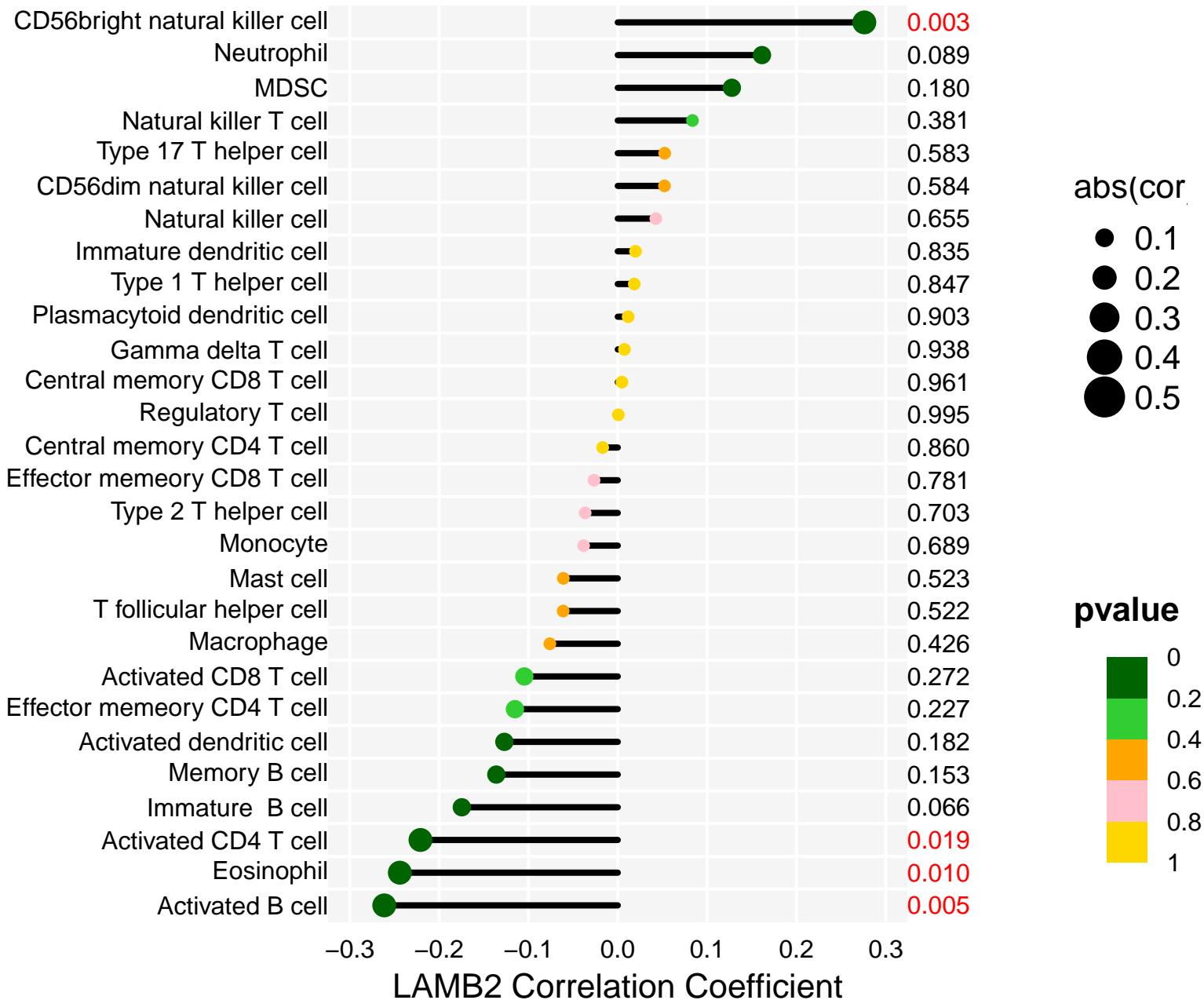

Supplement: Supplementary file 1 — Additional file 1. [file 12890_2023_2699_MOESM1_ESM.zip › 12890_2023_2699_MOESM1_ESM/Fig S1E_ESM.pdf]

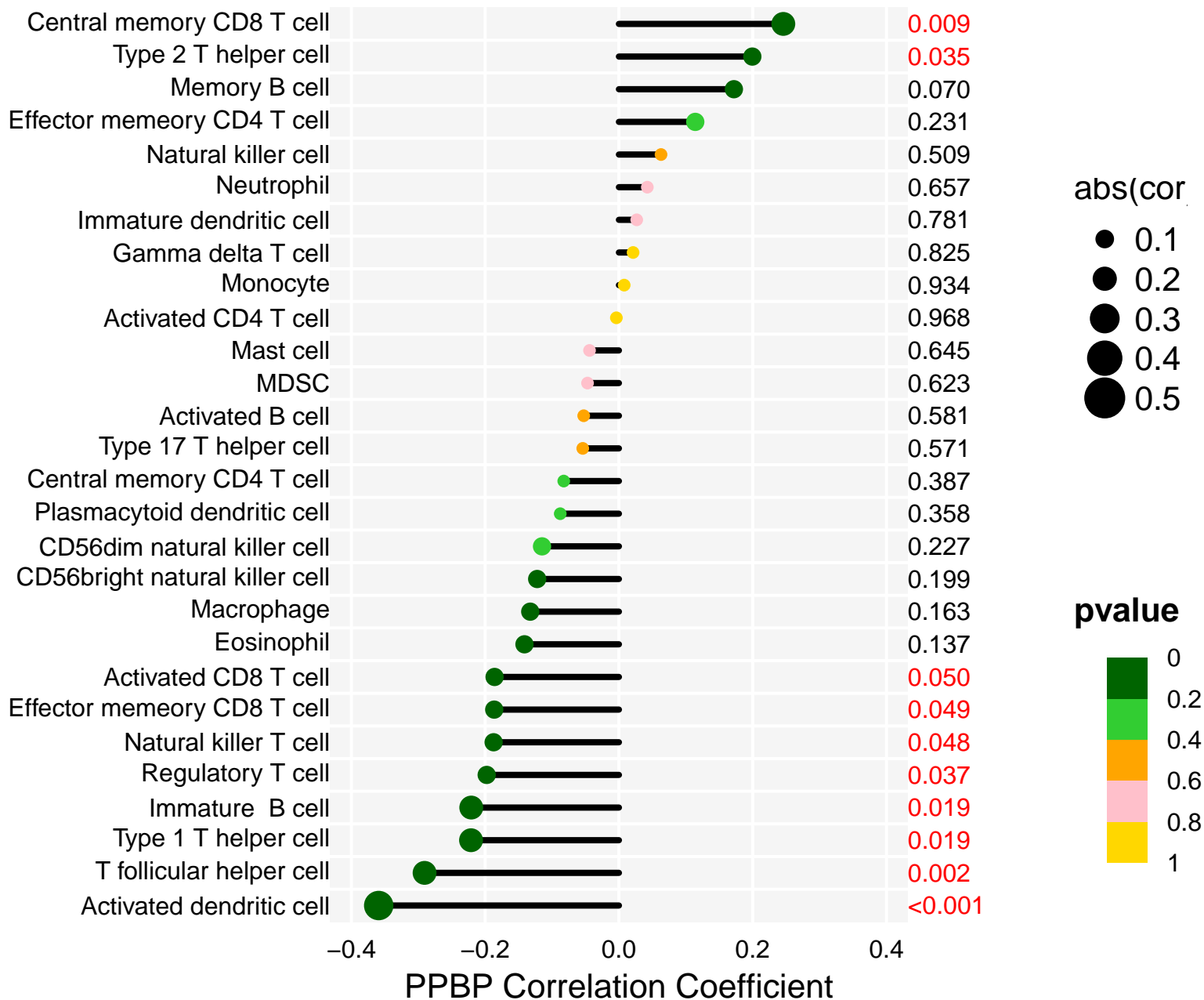

Supplement: Supplementary file 1 — Additional file 1. [file 12890_2023_2699_MOESM1_ESM.zip › 12890_2023_2699_MOESM1_ESM/Fig S1F_ESM.pdf]

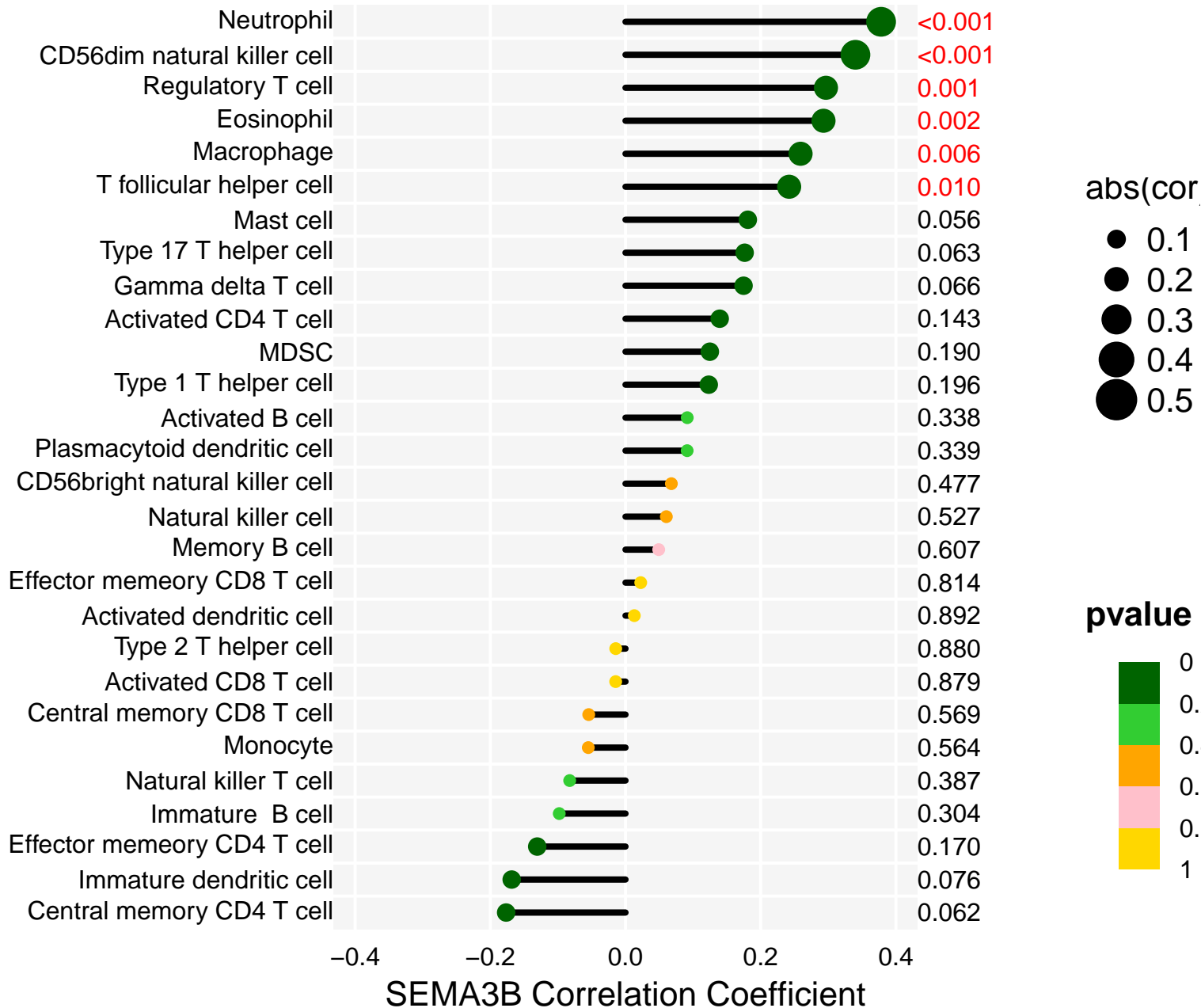

Supplement: Supplementary file 1 — Additional file 1. [file 12890_2023_2699_MOESM1_ESM.zip › 12890_2023_2699_MOESM1_ESM/Fig S1G_ESM.pdf]
